# Supplementary material for: Does environmental heterogeneity explain β diversity of estuarine fish assemblages? Example from a tropical estuary under the influence of a semiarid climate, Brazil
Source: PLoS One. 2022 Sep 22;17(9):e0273765. doi: 10.1371/journal.pone.0273765 (PMC9499227; doi:10.1371/journal.pone.0273765)
Supplement: S1 Appendix — (DOCX) [file pone.0273765.s001.docx]

Supporting information

S1 Appendix. List of fish species collected in the Mamanguape River estuary, Brazil. Frequency of Occurrence (O%), Numerical Percentage (N%), Biomass Percentage (%B) of species those were caught along three zones of Mamanguape river estuary during survey in 2015. Families were ordered according to Nelson (2006). TL – Total Length variation (Min-Max; mm). Guilds: Solely Estuarine (SE), Marine Straggler (MS), Marine Estuarine Opportunist (MEO), Marine Estuarine Dependent (MED), Estuarine & Marine (EM), Freshwater Straggler (FS), Freshwater Estuarine Opportunist (FEO).

|  |  | **Rainy** | | | | | | | | | | | |  | **Dry** | | | | | | | | | | |  |  |
| --- | --- | --- | --- | --- | --- | --- | --- | --- | --- | --- | --- | --- | --- | --- | --- | --- | --- | --- | --- | --- | --- | --- | --- | --- | --- | --- | --- |
| **Family/Species** |  | **guild** | **Zone 1** | | |  | **Zone 2** | | |  | **Zone 3** | | |  | **Zone 1** | | |  | **Zone 2** | | |  | **Zone 3** | | |  | **TL** |
|  |  |  | **O%** | **N%** | **W%** |  | **O%** | **N%** | **W%** |  | **O%** | **N%** | **W%** |  | **O%** | **N%** | **W%** |  | **O%** | **N%** | **W%** |  | **O%** | **N%** | **W%** |  | **Max-min** |
| **ELOPIDAE** |  |  |  |  |  |  |  |  |  |  |  |  |  |  |  |  |  |  |  |  |  |  |  |  |  |  |  |
| *Elops saurus* Linnaeus, 1766 |  | **MED** |  |  |  |  |  |  |  |  |  |  |  |  |  |  |  |  |  |  |  |  | 30.8 | 0.65 | 1.87 |  | 77-157 |
| *Elops saurus –* Leptocephalus Larvae |  | **MED** | 4 | 0.05 | <0.01 |  | 8 | 0.03 | <0.01 |  | 7.4 | 0.2 | <0.01 |  |  |  |  |  |  |  |  |  | 3.84 | 0.06 | <0.01 |  | 16-32 |
| **MEGALOPIDAE** |  |  |  |  |  |  |  |  |  |  |  |  |  |  |  |  |  |  |  |  |  |  |  |  |  |  |  |
| *Megalops atlanticus -* Leptocephalus Larva |  | **MED** |  |  |  |  |  |  |  |  | 7.4 | 0.18 | 0.03 |  |  |  |  |  |  |  |  |  | 3.84 | 0.09 | <0.01 |  | 25-33 |
| **ALBULIDAE** |  |  |  |  |  |  |  |  |  |  |  |  |  |  |  |  |  |  |  |  |  |  |  |  |  |  |  |
| *Albula vulpes* - Leptocephalus Larva |  | **MED** |  |  |  |  |  |  |  |  |  |  |  |  |  |  |  |  |  |  |  |  |  |  |  |  |  |
| **MORINGUIDAE** |  |  |  |  |  |  |  |  |  |  |  |  |  |  |  |  |  |  |  |  |  |  |  |  |  |  |  |
| *Moringua edwardsi* (Jordan & Bollman, 1889) |  | **MEO** |  |  |  |  | 8 | 0.03 | 0.06 |  |  |  |  |  |  |  |  |  | 3.7 | 0.04 | 0.04 |  |  |  |  |  | 144-161 |
| **OPHICHTHIDAE** |  |  |  |  |  |  |  |  |  |  |  |  |  |  |  |  |  |  |  |  |  |  |  |  |  |  |  |
| *Myrichthys ocellatus* (Lesueur, 1825) |  | **SE** |  |  |  |  |  |  |  |  |  |  |  |  |  |  |  |  |  |  |  |  | 3.84 | 0.03 | 0.26 |  | 390 |
| **ENGRAULIDAE** |  |  |  |  |  |  |  |  |  |  |  |  |  |  |  |  |  |  |  |  |  |  |  |  |  |  |  |
| *Anchoa hepsetus* (Linnaeus, 1758) |  | **MED** |  |  |  |  | 4 | 0.05 | 0.01 |  | 11.1 | 0.87 | 0.38 |  |  |  |  |  |  |  |  |  | 19.2 | 4.16 | 2.25 |  | 27-97 |
| *Anchoa januaria* (Steindachner, 1879) |  | **MED** | 12 | 0.2 | 0.08 |  | 20 | 0.52 | 0.16 |  | 3.7 | 0.02 | <0.01 |  |  |  |  |  |  |  |  |  | 26.9 | 5.47 | 1.32 |  | 25-68 |
| *Anchoa lyolepis* (Evermann & Marsh, 1900) |  | **MED** |  |  |  |  | 8 | 0.09 | 0.01 |  | 3.7 | 0.02 | <0.01 |  |  |  |  |  |  |  |  |  | 3.84 | 0.03 | 0.02 |  | 27-70 |
| *Anchoa marinii* Hildebrand, 1943 |  | **MED** | 4 | 0.05 | 0.07 |  | 12 | 0.17 | 0.04 |  |  |  |  |  |  |  |  |  |  |  |  |  | 7.69 | 1.08 | 0.3 |  | 25-66 |
| *Anchoa* sp. |  | **MED** | 4 | 0.05 | <0.01 |  | 20 | 0.39 | 0.04 |  | 11.1 | 17.8 | 1 |  |  |  |  |  | 3.7 | 0.63 | 0.07 |  | 30.8 | 1.21 | 0.07 |  | 39-51 |
| *Anchoa spinifer* (Valenciennes, 1848) |  | **MED** | 8 | 0.25 | 0.24 |  | 16 | 0.17 | 0.08 |  | 3.7 | 0.04 | 0.02 |  |  |  |  |  |  |  |  |  |  |  |  |  | 46-71 |
| *Anchoa tricolor* (Spix & Agassiz, 1829) |  | **MED** | 4 | 0.05 | 0.01 |  | 8 | 0.15 | 0.01 |  | 22.2 | 0.14 | 0.07 |  |  |  |  |  |  |  |  |  | 3.84 | 0.03 | <0.01 |  | 22-92 |
| *Anchovia clupeoides* (Swainson, 1839) |  | **MED** |  |  |  |  |  |  |  |  |  |  |  |  |  |  |  |  |  |  |  |  | 11.5 | 0.52 | 2.28 |  | 120-146 |
| *Anchoviella brevirostris* (Günther, 1868) |  | **MED** | 4 | 1.63 | <0.01 |  | 16 | 0.21 | 0.05 |  | 25.9 | 0.54 | 0.04 |  |  |  |  |  |  |  |  |  | 11.5 | 0.26 | 0.03 |  | 24-54 |
| *Anchoviella lepidentostole* (Fowler, 1911) |  | **MED** | 4 | 0.1 | 0.03 |  | 20 | 0.54 | 0.05 |  | 22.2 | 1.05 | 0.12 |  |  |  |  |  |  |  |  |  | 15.4 | 2.42 | 0.34 |  | 22-56 |
| *Anchoviella* sp. |  | **MED** | 12 | 0.35 | 0.08 |  | 12 | 0.29 | 0.06 |  | 18.5 | 1.17 | 0.08 |  | 3.7 | 0.1 | <0.01 |  | 3.7 | 0.09 | 0.01 |  | 15.4 | 3.08 | 0.13 |  | 21-45 |
| *Cetengraulis edentulus* (Cuvier, 1829) |  | **MED** |  |  |  |  |  |  |  |  |  |  |  |  |  |  |  |  |  |  |  |  | 3.84 | 0.03 | 0.02 |  | 74 |
| Engraulidae - Larva |  |  | 36 | 16.4 | 0.72 |  | 52 | 59.3 | 1.55 |  | 37 | 3.66 | 0.19 |  | 3.7 | 0.1 | <0.01 |  | 3.7 | 0.04 | <0.01 |  | 15.4 | 0.22 | <0.01 |  | 10-58 |
| *Lycengraulis grossidens* (Spix & Agassiz, 1829) |  | **MED** |  |  |  |  | 4 | 0.44 | 0.11 |  | 29.6 | 0.42 | 0.43 |  |  |  |  |  | 7.4 | 0.29 | 0.07 |  | 53.8 | 5.99 | 3.27 |  | 25-189 |
| **CLUPEIDAE** |  |  |  |  |  |  |  |  |  |  |  |  |  |  |  |  |  |  |  |  |  |  |  |  |  |  |  |
| *Harengula clupeola* (Cuvier, 1789) |  | **MED** |  |  |  |  |  |  |  |  |  |  |  |  |  |  |  |  |  |  |  |  | 23.1 | 4.68 | 4.5 |  | 41-89 |
| *Lile piquitinga* (Schreiner & Miranda Ribeiro, 1903) |  | **MED** |  |  |  |  |  |  |  |  | 7.4 | 0.28 | 1.16 |  |  |  |  |  |  |  |  |  |  |  |  |  | 71-107 |
| *Opisthonema oglinum* (Lesueur, 1818) |  | **MEO** |  |  |  |  |  |  |  |  |  |  |  |  |  |  |  |  |  |  |  |  | 7.69 | 0.09 | 0.04 |  | 71-76 |
| *Rhinosardinia bahiensis* (Steindachner, 1879) |  | **MED** | 12 | 1.88 | 5.25 |  | 36 | 0.48 | 0.05 |  | 29.6 | 1.72 | 0.4 |  |  |  |  |  | 3.7 | 0.04 | 0.01 |  | 26.9 | 9.11 | 7.87 |  | 15-95 |
| *Sardinella brasiliensis* (Steindachner, 1879) |  | **MEO** |  |  |  |  |  |  |  |  | 11.1 | 0.36 | 1.43 |  |  |  |  |  |  |  |  |  |  |  |  |  | 81-105 |
| **CHARACIDAE** |  |  |  |  |  |  |  |  |  |  |  |  |  |  |  |  |  |  |  |  |  |  |  |  |  |  |  |
| *Astyanax bimaculatus* (Linnaeus, 1758) |  | **FS** | 4 | 0.05 | 0.05 |  |  |  |  |  |  |  |  |  |  |  |  |  |  |  |  |  |  |  |  |  | 45 |
| *Astyanax fasciatus* (Cuvier, 1819) |  | **FS** |  |  |  |  |  |  |  |  |  |  |  |  | 3.7 | 0.1 | 0.1 |  |  |  |  |  |  |  |  |  | 56 |
| *Bryconamericus* sp. |  | **FS** | 4 | 0.05 | 0.01 |  |  |  |  |  |  |  |  |  |  |  |  |  |  |  |  |  |  |  |  |  | 32 |
| **ARIIDAE** |  |  |  |  |  |  |  |  |  |  |  |  |  |  |  |  |  |  |  |  |  |  |  |  |  |  |  |
| *Cathorops arenatus* (Valenciennes, 1840) |  | **SE** | 4 | 0.05 | 2.36 |  |  |  |  |  |  |  |  |  |  |  |  |  |  |  |  |  |  |  |  |  | 167 |
| *Sciades herzbergii* (Bloch, 1794) |  | **SE** | 28 | 20.4 | 36.3 |  | 4 | 0.03 | 0.05 |  | 7.4 | 0.04 | 0.01 |  |  |  |  |  | 3.7 | 0.04 | 0.04 |  |  |  |  |  | 31-94 |
| **SYNODONTIDAE** |  |  |  |  |  |  |  |  |  |  |  |  |  |  |  |  |  |  |  |  |  |  |  |  |  |  |  |
| *Synodus foetens* (Linnaeus, 1766) |  | **MEO** |  |  |  |  |  |  |  |  |  |  |  |  |  |  |  |  |  |  |  |  | 23.1 | 0.19 | 0.08 |  | 48-83 |
| **BATRACHOIDIDAE** |  |  |  |  |  |  |  |  |  |  |  |  |  |  |  |  |  |  |  |  |  |  |  |  |  |  |  |
| *Thalassophryne nattereri* Steindachner, 1876 |  | **SE** |  |  |  |  | 4 | 0.01 | 1.73 |  |  |  |  |  |  |  |  |  |  |  |  |  | 3.84 | 0.03 | <0.01 |  | 20-166 |
| **MUGILIDAE** |  |  |  |  |  |  |  |  |  |  |  |  |  |  |  |  |  |  |  |  |  |  |  |  |  |  |  |
| *Mugil brevirostris* (Ribeiro, 2015) |  | **MED** | 28 | 1.27 | 1.83 |  | 40 | 2.14 | 1.86 |  | 33.3 | 5 | 1.3 |  | 29.6 | 6.55 | 1.36 |  | 14.8 | 1.96 | 0.67 |  | 30.8 | 1.24 | 1.99 |  | 16-115 |
| *Mugil curema* Valenciennes, 1836 |  | **MED** | 12 | 1.88 | 0.68 |  | 36 | 0.39 | 2.09 |  | 25.9 | 0.75 | 0.54 |  | 3.7 | 1.63 | 0.38 |  | 18.5 | 1.96 | 0.55 |  | 7.69 | 0.16 | 0.32 |  | 18-200 |
| *Mugil curvidens* Valenciennes, 1836 |  | **MED** |  |  |  |  |  |  |  |  | 3.7 | 0.02 | 1.1 |  |  |  |  |  |  |  |  |  |  |  |  |  | 21 |
| *Mugil incilis* Hancock, 1830 |  | **MED** |  |  |  |  | 8 | 0.03 | <0.01 |  | 3.7 | 0.02 | <0.01 |  |  |  |  |  |  |  |  |  |  |  |  |  | 30-92 |
| *Mugil liza* Valenciennes, 1836 |  | **MED** |  |  |  |  | 12 | 0.5 | 0.06 |  | 3.7 | 0.06 | 0.03 |  | 7.4 | 0.3 | 0.41 |  | 3.7 | 0.68 | 0.11 |  | 7.69 | 0.16 | 0.12 |  | 18-72 |
| *Mugil rubrioculus* Harrison, Oliveira & Gaviria, 2007 |  | **MED** | 4 | 1.27 | 0.23 |  | 8 | 0.03 | 0.11 |  | 7.4 | 0.06 | 0.15 |  |  |  |  |  |  |  |  |  |  |  |  |  | 29-72 |
| *Mugil* sp. |  | **MED** |  |  |  |  | 4 | 0.03 | <0.01 |  | 3.7 | 0.34 | 0.01 |  |  |  |  |  |  |  |  |  |  |  |  |  | 16-32 |
| **ATHERONOPSIDAE** |  |  |  |  |  |  |  |  |  |  |  |  |  |  |  |  |  |  |  |  |  |  |  |  |  |  |  |
| *Atherinella blackburni* (Schultz, 1949) |  | **MEO** |  |  |  |  |  |  |  |  | 3.7 | 0.02 | <0.01 |  |  |  |  |  |  |  |  |  | 19.2 | 0.78 | 0.33 |  | 15-78 |
| *Atherinella brasiliensis* (Quoy & Gaimard, 1825) |  | **EM** | 72 | 14.7 | 23.8 |  | 80 | 16.6 | 38.5 |  | 88.9 | 19.7 | 21.4 |  | 92.6 | 58.1 | 45.6 |  | 96.3 | 67.6 | 56.8 |  | 76.9 | 26.6 | 24.6 |  | 10-131 |
| **HEMIRAMPHIDAE** |  |  |  |  |  |  |  |  |  |  |  |  |  |  |  |  |  |  |  |  |  |  |  |  |  |  |  |
| *Hyporhamphus roberti* (Valenciennes, 1847) |  | **EM** | 4 | 0.1 | 0.15 |  | 8 | 0.23 | 0.53 |  |  |  |  |  | 14.8 | 0.4 | 1.17 |  | 3.7 | 0.04 | 0.15 |  |  |  |  |  | 81-161 |
| *Hyporhamphus* sp. |  | **EM** |  |  |  |  |  |  |  |  |  |  |  |  |  |  |  |  |  |  |  |  | 3.84 | 0.06 | <0.01 |  | 30-32 |
| *Hyporhamphus unifasciatus* (Ranzani, 1841) |  | **EM** | 24 | 5.24 | 12 |  | 44 | 1.09 | 2.25 |  | 51.9 | 2.12 | 2.56 |  | 29.6 | 7.26 | 12.3 |  | 48.1 | 1.91 | 2.98 |  | 50 | 2.13 | 0.98 |  | 34-191 |
| **BELONIDAE** |  |  |  |  |  |  |  |  |  |  |  |  |  |  |  |  |  |  |  |  |  |  |  |  |  |  |  |
| Belonidae - Larva |  |  |  |  |  |  |  |  |  |  |  |  |  |  |  |  |  |  |  |  |  |  | 3.84 | 0.03 | <0.01 |  | 15 |
| *Strongylura marina* (Walbaum, 1792) |  | **EM** |  |  |  |  | 4 | 0.03 | 0.21 |  |  |  |  |  | 3.7 | 0.2 | 0.53 |  | 7.4 | 0.09 | 0.28 |  |  |  |  |  | 143-200 |
| *Strongylura* sp. |  | **EM** |  |  |  |  |  |  |  |  |  |  |  |  |  |  |  |  |  |  |  |  | 3.84 | 0.03 | <0.01 |  | 35 |
| *Strongylura timucu* (Walbaum, 1792) |  | **EM** | 20 | 0.45 | 1.23 |  | 8 | 0.03 | 0.07 |  | 11.1 | 0.06 | 0.13 |  | 18.5 | 1.12 | 0.94 |  | 18.5 | 0.34 | 0.87 |  | 23.1 | 0.22 | 0.16 |  | 41-232 |
| **POECIILIDAE** |  |  |  |  |  |  |  |  |  |  |  |  |  |  |  |  |  |  |  |  |  |  |  |  |  |  |  |
| *Poecilia vivipara* Bloch & Schneider, 1801 |  | **FEO** | 20 | 4.12 | 0.7 |  |  |  |  |  |  |  |  |  |  |  |  |  |  |  |  |  |  |  |  |  | 20-60 |
| **SYNGNATHIIDAE** |  |  |  |  |  |  |  |  |  |  |  |  |  |  |  |  |  |  |  |  |  |  |  |  |  |  |  |
| *Cosmocampus elucens* (Poey, 1868) |  | **SE** |  |  |  |  |  |  |  |  | 3.7 | 0.02 | <0.01 |  |  |  |  |  |  |  |  |  | 3.84 | 0.06 | <0.01 |  | 66-100 |
| *Pseudophalus mindii* (Meek & Hildebrand, 1923) |  | **SE** |  |  |  |  |  |  |  |  |  |  |  |  |  |  |  |  |  |  |  |  | 3.84 | 0.03 | <0.01 |  | 48 |
| *Syngnathus pelagicus* Linnaeus, 1758 |  | **SE** |  |  |  |  |  |  |  |  | 3.7 | 0.02 | <0.01 |  |  |  |  |  |  |  |  |  | 15.4 | 0.45 | 0.01 |  | 13-77 |
| **FISTULARIIDAE** |  |  |  |  |  |  |  |  |  |  |  |  |  |  |  |  |  |  |  |  |  |  |  |  |  |  |  |
| *Fistularia tabacaria* Linnaeus, 1758 |  | **MS** |  |  |  |  |  |  |  |  |  |  |  |  |  |  |  |  |  |  |  |  | 3.84 | 0.03 | 0.02 |  | 232 |
| **DACTYLOPTERIIDAE** |  |  |  |  |  |  |  |  |  |  |  |  |  |  |  |  |  |  |  |  |  |  |  |  |  |  |  |
| *Dactylopterus volitans* (Linnaeus, 1758) |  | **MED** |  |  |  |  |  |  |  |  | 7.4 | 0.12 | 0.46 |  |  |  |  |  |  |  |  |  | 3.84 | 0.03 | 0.02 |  | 36-124 |
| **CENTROPOMIDAE** |  |  |  |  |  |  |  |  |  |  |  |  |  |  |  |  |  |  |  |  |  |  |  |  |  |  |  |
| *Centropomus parallelus* Poey, 1860 |  | **MED** | 4 | 0.05 | 0.21 |  |  |  |  |  |  |  |  |  |  |  |  |  |  |  |  |  | 3.84 | 0.06 | <0.01 |  | 65-85 |
| *Centropomus undecimalis* (Bloch, 1792) |  | **MED** |  |  |  |  |  |  |  |  | 3.7 | 0.1 | 0.11 |  |  |  |  |  |  |  |  |  | 15.4 | 0.52 | 2.07 |  | 69-207 |
| **SERRANIDAE** |  |  |  |  |  |  |  |  |  |  |  |  |  |  |  |  |  |  |  |  |  |  |  |  |  |  |  |
| *Epinephelus marginatus* (Lowe, 1834) |  | **MEO** |  |  |  |  | 4 | 0.03 | <0.01 |  |  |  |  |  |  |  |  |  |  |  |  |  |  |  |  |  | 20-25 |
| *Serranus phoebe* Poey, 1851 |  | **MEO** |  |  |  |  |  |  |  |  |  |  |  |  |  |  |  |  |  |  |  |  | 3.84 | 0.03 | <0.01 |  | 18 |
| **CARANGIDAE** |  |  |  |  |  |  |  |  |  |  |  |  |  |  |  |  |  |  |  |  |  |  |  |  |  |  |  |
| *Caranx hippos* (Linnaeus, 1766) |  | **MED** |  |  |  |  |  |  |  |  |  |  |  |  |  |  |  |  |  |  |  |  | 11.5 | 0.26 | 0.68 |  | 57-111 |
| *Caranx latus* Agassiz, 1831 |  | **MED** | 16 | 0.4 | 1.79 |  | 60 | 0.74 | 1.56 |  | 81.5 | 7.42 | 6.73 |  | 3.7 | 0.1 | 0.2 |  | 7.4 | 0.09 | 0.19 |  | 65.4 | 4.39 | 5.7 |  | 30-122 |
| *Chloroscombrus chrysurus* (Linnaeus, 1766) |  | **MED** |  |  |  |  |  |  |  |  | 3.7 | 0.02 | <0.01 |  |  |  |  |  |  |  |  |  |  |  |  |  | 21 |
| *Oligoplites palometa* (Cuvier, 1832) |  | **MED** | 8 | 0.1 | 0.17 |  | 16 | 0.09 | 0.01 |  | 7.4 | 0.04 | <0.01 |  | 14.8 | 0.51 | 0.29 |  | 11.1 | 0.73 | 0.2 |  |  |  |  |  | 17-82 |
| *Oligoplites saurus* (Bloch & Schneider, 1801) |  | **MED** | 8 | 0.1 | 0.34 |  | 8 | 0.03 | 0.05 |  | 25.9 | 0.3 | 0.28 |  | 11.1 | 0.81 | 0.12 |  | 7.4 | 0.09 | 0.08 |  | 0.68 | 0.29 |  |  | 20-130 |
| *Selene vomer* (Linnaeus, 1758) |  | **MEO** |  |  |  |  |  |  |  |  |  |  |  |  |  |  |  |  |  |  |  |  | 19.2 | 0.16 | 0.14 |  | 43-85 |
| *Trachinotus falcatus* (Linnaeus, 1758) |  | **MEO** |  |  |  |  |  |  |  |  | 3.7 | 0.04 | 0.01 |  |  |  |  |  |  |  |  |  | 7.69 | 0.06 | 0.12 |  | 25-92 |
| **LUTJANIDAE** |  |  |  |  |  |  |  |  |  |  |  |  |  |  |  |  |  |  |  |  |  |  |  |  |  |  |  |
| *Lutjanus analis* (Cuvier, 1828) |  | **MED** | 4 | 0.05 | <0.01 |  |  |  |  |  | 7.4 | 0.1 | 0.25 |  |  |  |  |  |  |  |  |  | 7.69 | 0.13 | 0.46 |  | 20-130 |
| *Lutjanus cyanopterus* (Cuvier, 1828) |  | **MED** | 8 | 0.1 | 0.17 |  |  |  |  |  | 7.4 | 0.06 | <0.01 |  |  |  |  |  |  |  |  |  | 7.69 | 0.06 | 0.01 |  | 20-80 |
| *Lutjanus griseus* (Linnaeus, 1758) |  | **MED** |  |  |  |  |  |  |  |  |  |  |  |  |  |  |  |  |  |  |  |  | 3.84 | 0.09 | 0.01 |  | 14-37 |
| *Lutjanus jocu* (Bloch & Schneider, 1801) |  | **MED** |  |  |  |  |  |  |  |  |  |  |  |  |  |  |  |  | 3.7 | 0.04 | 0.34 |  |  |  |  |  | 96 |
| *Lutjanus synagris* (Linnaeus, 1758) |  | **MED** |  |  |  |  |  |  |  |  | 11.1 | 0.1 | 0.32 |  |  |  |  |  |  |  |  |  | 15.4 | 0.49 | 0.16 |  | 12-116 |
| **GERREIDAE** |  |  |  |  |  |  |  |  |  |  |  |  |  |  |  |  |  |  |  |  |  |  |  |  |  |  |  |
| *Diapterus auratus* Ranzani, 1842 |  | **MED** | 12 | 0.2 | 0.5 |  | 24 | 0.44 | 0.78 |  | 7.4 | 0.06 | 0.03 |  | 7.4 | 1.22 | 0.66 |  | 25.9 | 2.31 | 3.09 |  | 26.9 | 0.65 | 0.26 |  | 20-83 |
| *Diapterus rhombeus* (Cuvier, 1829) |  | **MED** |  |  |  |  | 28 | 0.66 | 0.89 |  | 3.7 | 0.02 | 0.13 |  | 3.7 | 0.1 | <0.01 |  | 7.4 | 0.24 | 0.22 |  | 15.4 | 0.16 | 0.07 |  | 17-82 |
| *Eucinostomus argenteus* Baird & Girard, 1855 |  | **MED** | 28 | 1.73 | 1.02 |  | 48 | 1.09 | 5.75 |  | 22.2 | 0.24 | 0.63 |  | 44.4 | 4.91 | 13.3 |  | 51.9 | 5.45 | 8.9 |  | 38.5 | 1.99 | 1.74 |  | 13-143 |
| *Eucinostomus melanopterus* (Bleeker, 1863) |  | **MED** | 56 | 9.88 | 2.56 |  | 76 | 3.17 | 1.09 |  | 74.1 | 5.67 | 1.54 |  | 37 | 2.96 | 1.86 |  | 40.7 | 2.8 | 4.31 |  | 38.5 | 0.58 | 0.41 |  | 11-111 |
| *Eugerres brasilianus* (Cuvier, 1830) |  | **MED** |  |  |  |  | 12 | 0.25 | 0.1 |  | 3.7 | 0.04 | 0.08 |  |  |  |  |  | 11.1 | 0.19 | 0.07 |  | 3.84 | 0.03 | <0.01 |  | 17-75 |
| Gerreidae - Larva |  |  | 12 | 10.3 | 0.69 |  | 44 | 1.13 | 0.05 |  | 77.8 | 21.1 | 0.25 |  | 7.4 | 0.3 | <0.01 |  | 14.8 | 0.49 | <0.01 |  | 23.1 | 0.88 | 0.01 |  | 07-36 |
| *Ulaema lefroyi* (Goode, 1874) |  | **MED** |  |  |  |  | 8 | 0.03 | 0.19 |  | 14.8 | 0.7 | 1.13 |  |  |  |  |  | 11.1 | 0.49 | 0.74 |  | 34.6 | 5.63 | 4.13 |  | 15-110 |
| **HAEMULIDAE** |  |  |  |  |  |  |  |  |  |  |  |  |  |  |  |  |  |  |  |  |  |  |  |  |  |  |  |
| *Haemulopsys corvinaeformis* (Steindachner, 1868) |  | **MED** |  |  |  |  |  |  |  |  | 3.7 | 0.04 | 0.47 |  |  |  |  |  |  |  |  |  | 23.1 | 1.76 | 3.27 |  | 13-132 |
| *Orthopristis rubra* (Cuvier, 1830) |  | **MED** | 4 | 0.05 | 0.01 |  |  |  |  |  |  |  |  |  |  |  |  |  |  |  |  |  |  |  |  |  | 135 |
| *Pomadasys ramosus* (Poey, 1860) |  | **MED** |  |  |  |  |  |  |  |  |  |  |  |  | 3.7 | 0.1 | 0.27 |  |  |  |  |  |  |  |  |  | 78 |
| **POLYNEMIDAE** |  |  |  |  |  |  |  |  |  |  |  |  |  |  |  |  |  |  |  |  |  |  |  |  |  |  |  |
| *Polydactylus virginicus* (Linnaeus, 1758) |  | **MED** |  |  |  |  |  |  |  |  | 7.4 | 0.04 | 0.38 |  |  |  |  |  |  |  |  |  | 3.84 | 0.03 | 0.16 |  | 47-129 |
| **SCIAENIDAE** |  |  |  |  |  |  |  |  |  |  |  |  |  |  |  |  |  |  |  |  |  |  |  |  |  |  |  |
| *Bairdiella ronchus* (Cuvier, 1830) |  | **MED** |  |  |  |  |  |  |  |  |  |  |  |  |  |  |  |  |  |  |  |  | 3.84 | 0.03 | <0.01 |  | 16 |
| *Menticirrhus littoralis* (Holbrook, 1847) |  | **MEO** |  |  |  |  |  |  |  |  |  |  |  |  |  |  |  |  |  |  |  |  | 7.69 | 0.06 | 1.36 |  | 122-228 |
| *Ophioscion punctatissimus* Meek & Hildebrand, 1925 |  | **MED** |  |  |  |  |  |  |  |  | 3.7 | 0.02 | 0.01 |  |  |  |  |  |  |  |  |  |  |  |  |  | 53 |
| *Stellifer rastrifer* (Jordan, 1889) |  | **MED** |  |  |  |  | 8 | 0.11 | <0.01 |  |  |  |  |  |  |  |  |  |  |  |  |  |  |  |  |  | 13-71 |
| **MULLIDAE** |  |  |  |  |  |  |  |  |  |  |  |  |  |  |  |  |  |  |  |  |  |  |  |  |  |  |  |
| *Pseudupeneus maculatus* (Bloch, 1793) |  | **MED** |  |  |  |  |  |  |  |  | 3.7 | 0.02 | <0.01 |  |  |  |  |  |  |  |  |  |  |  |  |  | 44 |
| **CICHLIDAE** |  |  |  |  |  |  |  |  |  |  |  |  |  |  |  |  |  |  |  |  |  |  |  |  |  |  |  |
| *Coptodon rendalli* (Boulenger, 1897) |  | **FS** | 4 | 0.05 | <0.01 |  | 4 | 0.03 | <0.01 |  |  |  |  |  |  |  |  |  |  |  |  |  |  |  |  |  | 20-26 |
| **SCARIDAE** |  |  |  |  |  |  |  |  |  |  |  |  |  |  |  |  |  |  |  |  |  |  |  |  |  |  |  |
| *Nicholsina usta* (Valenciennes, 1840) |  | **MS** |  |  |  |  |  |  |  |  |  |  |  |  |  |  |  |  |  |  |  |  | 19.2 | 0.49 | 0.02 |  | 15-37 |
| **DACTYLOSCOPIDAE** |  |  |  |  |  |  |  |  |  |  |  |  |  |  |  |  |  |  |  |  |  |  |  |  |  |  |  |
| *Dactyloscopus crossotus* Starks, 1913 |  | **MS** |  |  |  |  |  |  |  |  | 3.7 | 0.02 | <0.01 |  |  |  |  |  |  |  |  |  |  |  |  |  | 25 |
| **ELEOTRIDAE** |  |  |  |  |  |  |  |  |  |  |  |  |  |  |  |  |  |  |  |  |  |  |  |  |  |  |  |
| *Eleotris pisonis* (Gmelin, 1789) |  | **SE** |  |  |  |  |  |  |  |  |  |  |  |  | 3.7 | 0.1 | 0.07 |  |  |  |  |  |  |  |  |  | 51 |
| *Erotelis smaragdus* (Valenciennes, 1837) |  | **SE** |  |  |  |  |  |  |  |  | 3.7 | 0.02 | <0.01 |  |  |  |  |  |  |  |  |  |  |  |  |  | 50 |
| **GOBIIDAE** |  |  |  |  |  |  |  |  |  |  |  |  |  |  |  |  |  |  |  |  |  |  |  |  |  |  |  |
| *Bathygobius soporator* (Valenciennes, 1837) |  | **SE** |  |  |  |  | 12 | 0.05 | 0.23 |  | 33.3 | 0.6 | 1.58 |  | 11.1 | 0.4 | 1.39 |  | 18.5 | 0.24 | 0.61 |  | 34.6 | 0.95 | 1.46 |  | 18-121 |
| *Ctenogobius boleosoma* (Jordan & Gilbert, 1882) |  | **SE** | 56 | 3.56 | 0.36 |  | 24 | 0.17 | 0.02 |  | 22.2 | 0.24 | 0.01 |  | 44.4 | 5.22 | 0.37 |  | 37 | 0.78 | 0.06 |  | 34.6 | 0.68 | 0.03 |  | 13-61 |
| *Ctenogobius shufeldti* (Jordan & Eigenmann, 1887) |  | **SE** | 4 | 0.1 | <0.01 |  |  |  |  |  |  |  |  |  |  |  |  |  |  |  |  |  |  |  |  |  | 18-24 |
| *Ctenogobius smaragdus* (Valenciennes, 1837) |  | **SE** |  |  |  |  |  |  |  |  |  |  |  |  |  |  |  |  |  |  |  |  | 0.03 | 0.02 |  |  | 76 |
| *Ctenogobius stigmaticus* (Poey, 1860) |  | **SE** |  |  |  |  |  |  |  |  | 3.7 | 0.02 | <0.01 |  |  |  |  |  |  |  |  |  | 0.03 | <0.01 |  |  | 45 |
| Gobiidae sp. |  |  |  |  |  |  |  |  |  |  |  |  |  |  | 3.7 | 0.1 | <0.01 |  |  |  |  |  |  |  |  |  | 15 |
| *Gobionellus oceanicus* (Pallas, 1770) |  | **SE** |  |  |  |  | 12 | 0.07 | 0.56 |  |  |  |  |  |  |  |  |  |  |  |  |  | 3.84 | 0.03 | 0.15 |  | 56-166 |
| *Gobionellus stomatus* Starks, 1913 |  | **SE** |  |  |  |  | 4 | 0.01 | 0.05 |  | 7.4 | 0.26 | 0.01 |  |  |  |  |  |  |  |  |  |  |  |  |  | 15-106 |
| Gobiidae - Larva |  |  | 4 | 0.05 | <0.01 |  |  |  |  |  | 3.7 | 0.04 | <0.01 |  |  |  |  |  |  |  |  |  |  |  |  |  | 11-13 |
| **EPHIPPIDAE** |  |  |  |  |  |  |  |  |  |  |  |  |  |  |  |  |  |  |  |  |  |  |  |  |  |  |  |
| *Chaetodipterus faber* (Broussonet, 1782) |  | **MED** |  |  |  |  |  |  |  |  | 7.4 | 0.04 | 0.02 |  |  |  |  |  |  |  |  |  | 11.5 | 0.13 | 0.1 |  | 27-45 |
| **SPHYRAENIDAE** |  |  |  |  |  |  |  |  |  |  |  |  |  |  |  |  |  |  |  |  |  |  |  |  |  |  |  |
| *Sphyraena barracuda* (Edwards, 1771) |  | **EM** |  |  |  |  | 4 | 0.01 | 0.13 |  | 3.7 | 0.02 | 1.07 |  |  |  |  |  |  |  |  |  |  |  |  |  | 120-260 |
| *Sphyraena guachancho* Cuvier, 1829 |  | **EM** |  |  |  |  |  |  |  |  | 3.7 | 0.02 | 0.15 |  |  |  |  |  |  |  |  |  |  |  |  |  | 140 |
| **CYCLOPSETTIDAE** |  |  |  |  |  |  |  |  |  |  |  |  |  |  |  |  |  |  |  |  |  |  |  |  |  |  |  |
| *Citharichthys arenaceus* Evermann & Marsh, 1900 |  | **MED** |  |  |  |  |  |  |  |  |  |  |  |  | 7.4 | 0.51 | 1.46 |  | 3.7 | 0.09 | <0.01 |  | 3.84 | 0.03 | 0.02 |  | 75-90 |
| *Citharichthys cornutus* (Günther, 1880) |  | **MS** |  |  |  |  |  |  |  |  | 3.7 | 0.02 | 0.02 |  |  |  |  |  |  |  |  |  |  |  |  |  | 66 |
| *Citharichthys macrops* Dresel, 1885 |  | **MED** | 24 | 0.71 | 0.1 |  | 28 | 0.54 | 0.09 |  | 11.1 | 0.06 | 0.15 |  | 7.4 | 0.2 | 0.09 |  | 14.8 | 0.24 | 0.04 |  | 7.69 | 0.06 | <0.01 |  | 10-94 |
| *Citharichthys spilopterus* Günther, 1862 |  | **MED** | 4 | 0.05 | <0.01 |  | 28 | 0.15 | 0.35 |  | 37 | 0.32 | 1.01 |  | 14.8 | 0.4 | 1.31 |  | 7.4 | 0.09 | 0.66 |  | 11.5 | 0.39 | 0.57 |  | 16-140 |
| *Etropus crossotus* Jordan & Gilbert, 1882 |  | **SE** |  |  |  |  |  |  |  |  | 3.7 | 0.04 | <0.01 |  |  |  |  |  |  |  |  |  |  |  |  |  | 25-28 |
| **ACHIRIDAE** |  |  |  |  |  |  |  |  |  |  |  |  |  |  |  |  |  |  |  |  |  |  |  |  |  |  |  |
| *Achirus declivis* Chabanaud, 1940 |  | **SE** | 4 | 0.2 | 0.44 |  | 4 | 0.03 | 0.2 |  |  |  |  |  |  |  |  |  |  |  |  |  |  |  |  |  | 46-91 |
| *Achirus lineatus* (Linnaeus, 1758) |  | **SE** | 36 | 0.81 | 0.83 |  | 40 | 0.54 | 2.73 |  | 7.4 | 0.08 | 0.11 |  | 48.1 | 3.37 | 3.06 |  | 70.4 | 3.83 | 4.68 |  | 23.1 | 0.22 | 0.82 |  | 11-95 |
| *Gymnachirus nudus* Kaup, 1858 |  | **MS** |  |  |  |  |  |  |  |  | 3.7 | 0.02 | <0.01 |  |  |  |  |  |  |  |  |  |  |  |  |  | 12 |
| *Trinectes microphthalmus* (Chabanaud, 1928) |  | **EM** |  |  |  |  |  |  |  |  |  |  |  |  | 7.4 | 0.2 | 0.11 |  | 0.04 | 0.01 |  |  |  |  |  |  | 31-42 |
| *Trinectes paulistanus* (Miranda Ribeiro, 1915) |  | **SE** |  |  |  |  | 20 | 2.22 | 4.21 |  |  |  |  |  | 3.7 | 0.1 | 0.02 |  |  |  |  |  |  |  |  |  | 18-99 |
| **CYNOGLOSSIDAE** |  |  |  |  |  |  |  |  |  |  |  |  |  |  |  |  |  |  |  |  |  |  |  |  |  |  |  |
| *Symphurus tesselatus* (Quoy & Gaimard, 1824) |  | **MED** |  |  |  |  | 8 | 0.09 | 1.75 |  | 7.4 | 0.06 | 0.65 |  |  |  |  |  |  |  |  |  | 3.84 | 0.13 | 0.53 |  | 38-175 |
| **TETRAODONTIDAE** |  |  |  |  |  |  |  |  |  |  |  |  |  |  |  |  |  |  |  |  |  |  |  |  |  |  |  |
| *Colomesus psittacus* (Bloch & Schneider, 1801) |  | **SE** | 4 | 0.05 | 0.73 |  |  |  |  |  |  |  |  |  | 3.7 | 0.1 | <0.01 |  |  |  |  |  |  |  |  |  | 15-82 |
| *Lagocephalus laevigatus* (Linnaeus, 1766) |  | **MEO** |  |  |  |  |  |  |  |  |  |  |  |  |  |  |  |  |  |  |  |  | 3.84 | 0.03 | 0.68 |  | 171 |
| *Sphoeroides greeleyi* Gilbert, 1900 |  | **MED** | 4 | 0.05 | 0.33 |  | 24 | 0.6 | 2.08 |  | 63 | 2.23 | 3.94 |  |  |  |  |  | 14.8 | 0.34 | 0.24 |  | 46.2 | 3.47 | 2.27 |  | 10-114 |
| *Sphoeroides* sp. |  |  |  |  |  |  |  |  |  |  |  |  |  |  |  |  |  |  | 3.7 | 0.04 | 0.08 |  |  |  |  |  | 57 |
| *Sphoeroides spengleri* (Bloch, 1785) |  | **MEO** |  |  |  |  |  |  |  |  | 7.4 | 0.06 | 0.05 |  |  |  |  |  |  |  |  |  | 23.1 | 0.88 | 0.07 |  | 15-73 |
| *Sphoeroides testudineus* (Linnaeus, 1758) |  | **SE** | 28 | 0.61 | 3.81 |  | 68 | 3.33 | 27.1 |  | 66.7 | 1.58 | 44.4 |  | 22.2 | 1.94 | 3.77 |  | 81.5 | 5.35 | 12.7 |  | 57.7 | 1.21 | 18.7 |  | 11-240 |
| **Species number** |  |  | **46** | | |  | **56** | | |  | **75** | | |  | **34** | | |  | **37** | | |  | **77** | | |  |  |
| **Individual number** |  |  | **1,963** | | |  | **5,127** | | |  | **4,932** | | |  | **977** | | |  | **2,034** | | |  | **3,051** | | |  |  |
| **Total biomass** |  |  | **1,913.69** | | |  | **6,091.19** | | |  | **9,503.67** | | |  | **1,634.56** | | |  | **3,452.27** | | |  | **9,793.13** | | |  |  |
