# Supplementary material for: Does environmental heterogeneity explain β diversity of estuarine fish assemblages? Example from a tropical estuary under the influence of a semiarid climate, Brazil
Source: PLoS One. 2022 Sep 22;17(9):e0273765. doi: 10.1371/journal.pone.0273765 (PMC9499227; doi:10.1371/journal.pone.0273765)
Supplement: Attachment — Submitted filename: renamed_e9538.docx [file pone.0273765.s003.docx]

Campina Grande, Brazil May 25^th^, 2022.

**To: Prof. Luiz Ubiratan Hepp – Academic Editor**

**Ref. Author's Response to Reviewer Comments (PONE-D-22-05724)**

Author's Response to Reviewer Comments

The authors would like to thank the area editor and the reviewer’s careful and thorough reading of this manuscript and for the thoughtful comments and constructive suggestions, which help to improve the quality of this manuscript. Please find below our responses to reviewer comments on our manuscript entitled "**Does environmental heterogeneity explain β diversity of estuarine fish assemblages? Example from a tropical estuary under the influence of a semiarid climate, Brazil**” submitted to  **PLOS ONE (PONE-D-22-05724)**. We responded to each comment in detail below and with the necessary revisions in the manuscript. As you will see, we addressed all suggestions and all of them were accepted and incorporated in this revised version.

On the behalf of the co-authors, I thank you again for your time and effort spent handling our manuscript.

Best regards,

André Luiz Machado Pessanha

|  | **# REVIEW 1**  Coauthors and I very much appreciated the encouraging, critical and constructive comments on this manuscript by the reviewer. The comments have been very thorough and useful in improving the manuscript. We strongly believe that the comments and suggestions have increased the scientific value of revised manuscript by many folds. |
| --- | --- |
| **Reviewer’s comment** | I strongly suggest you to include the list of all 125 species as a supplementary file |
| **Authors’ comment** | The species list was included as a supplementary file. |
|  |  |
| **Reviewer’s comment** | P1, L1: Very long title. Suggestion: Environmental heterogeneity from a tropical estuary under the influence of a semiarid climate, Brazil |
| **Authors’ comment** | We thank the reviewer for pointing this out. However, we decided to leave the original title because it provides information about which taxon we are studying. |
|  |  |
| **Reviewer’s comment** | Page 1, L4: It´s not necessary because there is just one Institution. |
| **Authors’ comment** | Changes made. |
|  |  |
| **Reviewer’s comment** | P1, L16: River. Revise all others. |
| **Authors’ comment** | Thank you for this point. The authors revised the suggested change from "river" to "River" throughout the text. |
|  |  |
| **Reviewer’s comment** | P1, L34: Do not use words already contained in the title. |
| **Authors’ comment** | The authors completely agree with reviewer comment on the sentence. Authors add the following keywords: Shallow habitats and Diversity partitioning . |
|  |  |
| **Reviewer’s comment** | P3; L86: Do not italicized it. Check "Instruction to authors" for all other similar cases. |
| **Authors’ comment** | We thank the reviewer for this observation; the problem has been fixed. |
|  |  |
| **Reviewer’s comment** | P5, L142: “available at [www.aesa.pb.gov.br](http://www.aesa.pb.gov.br).” This is cited in the References, it isn't necessary here. |
| **Authors’ comment** | We understand the reviewer's concern regarding the structure of the paper. Change made. |
|  |  |
| **Reviewer’s comment** | P9, L242: “A priori”. Do italicized it. |
| **Authors’ comment** | Changes made. |
|  |  |
| **Reviewer’s comment** | *P14, L 428: “The mojarra fish”* What family? |
| **Authors’ comment** | We appreciate the reviewer’s suggestion. We include the family in the text: “mojarra fish species (Gerreidae)” |
|  |  |
| **Reviewer’s comment** | *P14, L 431:* “two puffer fish species”. Could you to cite them? |
| **Authors’ comment** | We appreciate the reviewer’s suggestion. We include the two species in the text: “two puffer fish species (*S. testudineus* and *Colomesus psittacus*)” |
|  |  |
| **Reviewer’s comment** | P14, L435: Change “This result was possible” by “This result is due...” |
| **Authors’ comment** | Changes made. |
|  | **# REVIEW 2**  We thank Reviewer #2 for the constructive and insightful comments, which have helped us to substantially improve our manuscript. |
| **Reviewer’s comment** | Bibliographic references must be fully revised according to the journal's formatting rules. This includes citations in the body of the manuscript, which must be presented in numerical order. |
| **Authors’ comment** | We thank the reviewer for this observation; the problem has been fixed. |
|  |  |
| **Reviewer’s comment** | Some citations were not found in the references: line 50-Datry et al 2017; line 211-The R Development Core Team 2021 (see line 850-R Core Team, 2021); line 92-Anderson 2011; line 261-Baselga et al 2010; line 456-Clark et al 2014; 468-da Silva and Fabré 2019; 491-Garcia and Pessanha 2017; 528-da Silva and Fabré 2019. |
| **Authors’ comment** | The authors completely agree with reviewer comment on the sentence. Some citations were included (Datry et al 2017; da Silva and Fabré 2019), other revised (“Anderson 2011” by Anderson et al 2011; “The R Development Core Team 2021” by R Core Team, 2021; “Baselga et al 2010” by Baselga 2010; and others deleted (Clark et al 2014; Garcia and Pessanha 2017); |
|  |  |
| **Reviewer’s comment** | Some references were not found in the citations: lines 598; 601; 635; 682; 803; 881. |
| **Authors’ comment** | The citations Baselga 2012 (line 601), Camara et al 2019 (line 635), Gómez et al (2017) (line 682), Xu et al 2015 (line 881) were deleted. The citatiions Baselga et al (2010) and Potter et al (2015), lines 598 and 803 respectively, remained in the text. |
|  |  |
| **Reviewer’s comment** | The reference 655 is undated. |
| **Authors’ comment** | We thank the reviewer for this observation; the problem has been fixed. |
|  |  |
| **Reviewer’s comment** | Suggestion 1: when you describe diversity (2.5.3 line 235), you could use: "B1 corresponds to the variation of diversity between the sample subunits", as used in line 357. |
| **Authors’ comment** | Changes made. |
|  |  |
| **Reviewer’s comment** | Suggestion 2: Do not use the terms "semiarid" and "Tropical estuary" as keywords, as they are already included in the title of the manuscript. |
| **Authors’ comment** | The authors completely agree with reviewer comment on the sentence. Authors add the following keywords: Shallow habitats and Diversity partitioning . |
|  | **#Additional Editor Comments** |
| **Editor comment** | I believe that the time scale by the aforementioned authors refers to intra-annual (seasonal) |
| **Authors’ comment** | We thank the editor for this observation. In fact, the expression that should be used is “seasonal variation”. We made the modification to the entire text. |
|  |  |
| **Editor comment** | Another important point to what? in relation between two periods of the year What is the hypothesis for this? Are there waits for biological occurrences? |
| **Authors’ comment** | We thank the reviewer for his thoughtful and thorough review and believe his input has been invaluable to make our review more balanced. We have added the information in the hypothesis on seasonality in the revised manuscript. |
|  |  |
| **Editor comment** | I suggest a description of 'temporal variation' in the study, time scale is not replicated review the study properly. What we can observe are seasonal variables in an annual cycle. |
| **Authors’ comment** | We thank the editor for this observation. In fact, the expression that should be used is “seasonal variation”. We made the modification to the entire text. |
|  |  |
| **Editor comment** | As the authors test the features being land features as the landscape information is estuary scales, and this factor is not replicated. Explain. |
| **Authors’ comment** | We thank the editor for pointing this out. As in each zone of the estuary there were three sampling points, the measurements of channel width and distance from the mouth of the estuary were measured according to the location of these points. |
|  |  |
| **Editor comment** | Review the degrees of freedom for line 330. Are they correct? |
| **Authors’ comment** | The observations of reviewer are exact. Degrees of freedom was deleted in sentence. |
|  |  |
| **Editor comment** | I suggest that the authors review the criteria for typing errors and to meet the submission rules. |
| **Authors’ comment** | Changes made.  Again, we appreciate all of your insightful comments. We worked hard to be responsive to them. Thank you for taking the time and energy to help us improve the paper. |
|  |  |
